# Vaccination as a Control Tool in Bovine Tuberculosis: Social Media Monitoring to Assess Public Response to Government Policy Development and Implementation

**DOI:** 10.3390/vaccines9040314

**Published:** 2021-03-29

**Authors:** Frederika Dicks, Tatjana Marks, Emilie Karafillakis, Mark A Chambers

**Affiliations:** 1 School of Veterinary Medicine, VSM Building, University of Surrey, Daphne Jackson Road, Guildford, Surrey GU2 7AL, UK; fd00185@surrey.ac.xn; 2 Vaccine Confidence Project, London School of Hygiene & Tropical Medicine, Keppel St, Bloomsbury, London WC1E 7HT, UK; Tatjana.Marks2@student.lshtm.ac.uk (T.M.); Emilie.Karafillakis@lshtm.ac.uk (E.K.); 3 School of Biosciences and Medicine, Edward Jenner Building, University of Surrey, Guildford, Surrey GU2 7XH, UK

**Keywords:** badger, cattle, cull, media monitoring, policy, tuberculosis, vaccination

## Abstract

Vaccine hesitancy does not only concern human vaccines but incorporates One Health policies also; including vaccination of cattle and badgers as part of the government’s bovine tuberculosis eradication strategy for England. Both digital and social media can propagate healthcare misinformation and thus affect vaccine policy support. The use of social media monitoring to understand real-time public perceptions of One Health policies is crucial to identify misinformation and address public concerns appropriately to achieve successful policy implementation. Digital and social media data surrounding two government announcements regarding the bovine tuberculosis eradication strategy for England were collected and screened using the Meltwater media monitoring platform. Communication patterns were studied using InfraNodus. Twitter analysis was conducted to identify key influencers, public engagement, and trending communications. Online social media activity increased rapidly after each announcement. Initially, badger culling took primary public concern and major influencers were identified. Cattle vaccination dominated discussion after the second announcement, with public perception being influenced by increased online activity from news sites, animal welfare charities, governmental bodies, and medical professionals. The greatest ambiguity towards the strategy was detected within farming communities, with the main disparity existing between cattle vaccination and badger culling opinions. Social media monitoring has potential use in surveying public perception of government policy, both prior to, and after implementation to identify and address areas of miscommunication and misinformation to improve public support for One Health policies.

## 1. Introduction

The last twenty years have been a revolution for the internet, leading to the globalized access of vast amounts of information. Yet, with this increasing online access and the birth of a multitude of digital and social media platforms, the generation of misinformation has arisen. Distrust in the medical and government communities is not a novel, nor an isolated phenomenon [1]: confidence in the British public health authorities has peaked and troughed throughout the years, and unsurprisingly vaccination support has followed suit [2]. Whilst the birth of social and online media has enabled the spread of information regarding public health vaccine progress, the contagion and amplification of vaccination controversies online have become apparent. A prominent digital and social media issue is the notable rise and dissemination of unchecked, false information from both disreputable and in some cases reputable [3] sources, which is becoming an important dilemma for public health systems today [4]. One such dilemma that has arisen from such misinformation is that of vaccine hesitancy, with NHS childhood vaccination statistics for England 2018–2019 reporting coverage has declined in all routine vaccinations for children under five years old [5]. Vaccine hesitancy has also extended to animal vaccination settings, including canine Parvovirus vaccination [6], with increasing numbers of owners opting not to vaccinate their pets on the basis of misinformation and concerns. Considering the human-animal bond in many households, it is reasonable to draw comparison between the decline of human vaccination and that of companion animal vaccination.

Vaccination as a means of disease control has been part of livestock health management for decades and contributes enormous benefit both to the agricultural economy and public health. The implementation of an animal brucellosis vaccination program in Western Greece embodies this benefit, whereby incidence rates in both humans and animals decreased significantly [7]. Yet, despite numerous success stories, vaccination remains one of the most debated public health topics on social media platforms [8]. Agenda-setting theory states that content widely shared or trending in the news can impact public opinions by increasing the perceived saliency of specific issues [9]. Additionally, some media or news channels may also influence the areas of focus of other channels, referred to as intermedia agenda setting [10]. An unexplored question is to what extent and in what manner have digital and social media influenced opinion regarding livestock (and wildlife) vaccination? To address this question, we have used social listening tools to understand public opinion and perception of government policy for the intended control and eradication of bovine tuberculosis (bTB) from England. This is an especially pertinent example, since an iconic and protected wildlife species, the European badger (*Meles meles*), is implicated in transmission of the bacterium that causes bTB. It is generally recognized that elimination of the disease in cattle will be extremely difficult without measures to control the disease in badgers [11]. Government policy for England (as devised by the Department for Food and Rural Affairs (Defra) and ratified by the Secretary of State for Environment, Food and Rural Affairs) currently includes the highly contentious licensed culling and/or vaccination of badgers, combined with the routine test-and-slaughter of cattle [12]. Badger culling has been a controversial topic since its introduction in 2013, resulting in widespread media coverage. A 2014 YouGov survey of around 2000 adults in the UK suggested that whilst only 36% supported badger culling to reduce tuberculosis (TB) transmission, the remaining 64% determined it should not proceed or did not know. The poll revealed that 57% of participants believed the 2014 government handled the issue of TB in badgers and cattle poorly [13]. In March 2020, Defra outlined one of the biggest shifts in bTB control policy for decades; indicating the cessation of badger culling in favor of increased use of the licensed badger vaccine and a concerted effort to license a vaccine for use in cattle [14].

The primary objective of this study was to analyze online public attitudes and perceptions towards elements of a revised government strategy to achieve bTB-free status in England by 2038 [14]. We focused on two announcements (5 March 2020 and 22 July 2020) of the introduction of a bovine vaccination program using the Bacillus Calmette-Guérin (BCG) vaccine, the phasing out of badger culling, and increased use of badger vaccination. Digital and social media posts surrounding these announcements were evaluated and a comparative qualitative analysis was performed to identify structural gaps between the government policy statements and public understanding. Given our ‘online’ status as a society, it has been recognized that listening to both digital and social media conversations is paramount to gain public confidence and respond successfully to public concern [15]. Understanding public perception in real-time is crucial to relay appropriate and efficient responses to ensure a more thorough and uniform understanding of the government’s messages, and to debunk potential misinformation online. This was a vital analytical aspect of our study; providing insights into how communication gaps may be bridged between government announcements and the perceived messages understood by the public.

## 2. Materials and Methods

### 2.1. Data Capture Points

Digital and social media data were initially captured across a four-week period either side of a government announcement (5 March 2020), regarding the latest strategy for achieving bTB-free status for England (Annoucement-1) [14]. The statement was issued as the official government response to an independent review commissioned by Environment Secretary Michael Gove and led by Sir Charles Godfray in 2018 [16]. A second phase of data capture was conducted for the week surrounding the second government announcement (22 July 2020) to proceed with bTB vaccination field trials for cattle in England and Wales (Annoucement-2) [17]. Each digital and social media post was categorized (Table 1) and thematic content assessed to gauge either neutral, favorable, or unfavorable sentiments or opinions towards the latest bTB policy, focusing on the novel introduction of cattle vaccination trials.

### 2.2. Media Monitoring and Data Aggregation

**Figure 1:**
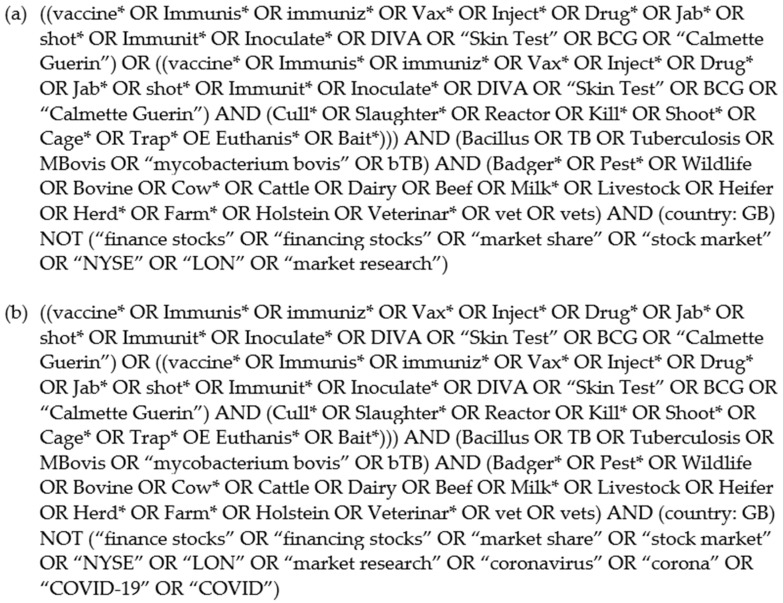
(**a**) Announcement-1 Boolean search. Created for Announcement-1 data collection on the Meltwater platform. This Boolean search was piloted initially to assess data collection prior to use; (**b**) Announcement-2 Boolean search, amended from Announcement-1 considering the surge in COVID-19-related digital and social media posts that were gathered during the first data collection.

Meltwater software (Meltwater: Media Monitoring and Social Listening Platform, www.meltwater.com/en, accessed on 14 April 2020) was used to collect and analyze digital and social media data within the two-week period prior to (pre-Announcement-1: 19 February to 4 March 2020), and the two weeks after (post-Announcement-1: 5 to 19 March 2020) Annoucement-1. The timely nature of Announcement-2 on 22 July 2020 provided an unforeseen follow-up dataset for analysis; data collection spanning the week surrounding this announcement (18 July 2020 to 26 July). A previously piloted Boolean search was used to aggregate data from social and digital media for Annoucement-1 (Figure 1a). An amended Boolean search was used for Annoucement-2 (Figure 1b), to remove coronavirus-related posts. Data gathered via the Meltwater software included several sources: the primary being Twitter (Twitter Inc., San Francisco, CA, USA), and the remainder being other social network, forum, and news sites. Subsequent analyses were carried out with the removal of direct news-site posts into a ‘neutral’ category to minimize media bias [18].

**Figure 2:**
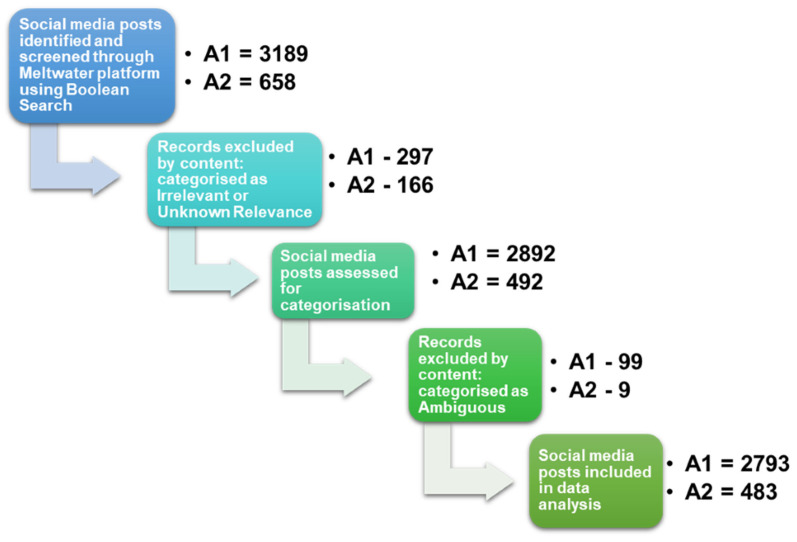
Flow diagram of the filtering process used to screen the digital and social media posts surrounding Annoucement-1 (A1) and Annoucement-2 (A2) collected from the Meltwater platform using the Boolean search. Posts that were excluded from the analysis were those categorized as irrelevant, unknown relevance and ambiguous. The ‘-’ sign in the figure represents the subtraction of the posts that were excluded from the analysis in each announcement. Remaining posts were analyzed and placed into one, or multiple of the seven categories: badger cull favorable or unfavorable, badger vaccine favorable or unfavorable, cattle vaccine favorable or unfavorable, or neutral.

The initial Meltwater search around Annoucement-1 generated a total of 3189 digital and social media posts (duplicates included) (Figure 2). Posts were screened by both author and content and categorized using the criteria listed in Table 1. The categories irrelevant, unknown relevance (*N* = 297) and ambiguous (*N* = 99) produced 396 digital and social media posts which were excluded from the analysis. The remaining 2793 digital and social media posts were included in further analysis.

Using Meltwater analytics (Meltwater.com, London, UK) and the primary Twitter analysis, data were further broken down to identify top words and hashtags, original tweets, retweets and quoted tweets, key influencers, and the extent of their social reach. A comparative Twitter analysis was performed to assess sentiments towards the two announcements of, first, the renewed bTB control strategies, and second, of the go-ahead for the cattle vaccination trials in England. Data were categorized by content into the following: (a) cattle vaccination; (b) badger vaccination; and (c) badger culling. To further evaluate digital and social media content from all data sources, four categories of favorable, unfavorable, ambiguous, and neutral (Table 1) were created, based on the main topics of discussion of posts as revealed by word frequency analysis using methodologies for vaccination stance analysis and categorization developed and described by Martin et al. [19]. Each digital and social media post was manually tagged by one researcher with either one or multiple categories, which were then downloaded as comma-separated values (CSV) files for further qualitative data analysis. To prevent analysis of online news articles, these media posts were separated out from the opinion analysis and placed directly into the ‘neutral’ category to provide a comparison. Any re-postings of said news articles that subjectively incorporated an opinionized message or viewpoint was included in the analysis on the basis that it was categorized as an opinion. The same method of data collection and analysis was employed for Announcement-2 by the same researcher. As only one researcher tagged the data, no inter-rater reliability procedure was performed.

Furthermore, qualitative analysis of all data sources involved subjective assessment and thematic analysis and characterization of digital and social media posts based on the written content and implied opinions regarding the words used and their relationships to set categories (Table 1). The categories aimed to decipher common themes and develop classes for similarities and variations of public opinion, upon which a numerical value of one was applied for each tagged category within the digital and social media posts. These values enabled clearer comparisons to be drawn between pre-announcement and post-announcement results to expose trends in content.

To protect users’ anonymity, data extracted from Meltwater was accessed by only one researcher and saved on a secured password-protected computer. Additionally, all files will be removed within two years of data collection. Personal identifiers of individuals such as names or Twitter handles were removed for data analysis and reporting. As this study only included publicly available data, we included data from organizations and public figures such as politicians in the analysis of key influencers.

### 2.3. Data Visualisation

InfraNodus (Nodus Labs, Berlin, Germany) (www.infranodus.com, accessed on 4 May 2020 is an open source visualization tool that enables the representation of text as a network of communication [20]. The objective was to identify communication changes within and between each announcement period. This included analysis of the most prominent words, hashtags, and authors (Twitter handles), notable links in communication clusters and prevalent themes (influential elements) from all data sources. After the digital and social media posts were categorized and tagged in Meltwater, the data were downloaded into individual categorized CSV files. The written content of each digital and social media post, including the author and any text quoted, were compiled to form the ‘hit sentences’ from each CSV file. These hit sentences were uploaded into InfraNodus to produce discrete categorized graphs. Content analysis was performed using the ‘Text Network Analysis’ tool to generate insight about the data that were linked and connected within and between the individual and overlapping categories. This network analysis focused on: the main topical groups and influential authors—referring to the frequency that a word or author connects different conversation clusters; the network structure—either focused or diversified; and the betweenness centrality—referring to how often words act as a bridge between other words. Words with the highest betweenness centrality are also termed ‘most influential elements’ and consider the impact of these words on the entire network [20].

InfraNodus data visualization enabled the quantification and identification of relationships of certain words, themes and ideas discussed within the digital and social media posts and was utilized to identify communication trends of either an individual personal or greater social nature. From these results, the aim was to reveal patterns within the communication content and identify any areas where there were gaps in the network. The network gaps represent areas of potential poor communication or topics that received reduced online attention (‘noise’) when compared to other aspects of the announcement that were ‘louder’ and well received and transferred in the onward chain of digital and social media. The aim of identifying these communication gaps was to propose their future use as a communication technique to create a bridging effect, to ensure a more uniform public reception and timely understanding of future government messages.

## 3. Results

### 3.1. Data Search and Categorisation

A total of 2793 digital and social media posts were included in the analysis (Figure 2), with many posts belonging in up to three categories, resulting in an overlap effect (Table 2). Relevant posts were further separated into pre- and post-announcement timeframes for Announcement-1 to conduct qualitative analysis. This included the identification of influencers and their social reach, hashtags, and public sentiments for a categorized data analysis. The follow-up Meltwater analysis for Announcement-2 produced a total of 658 digital and social media posts that were categorized according to Table 1. Upon removal of the 175 posts designated to irrelevant, unknown relevance and ambiguous, there were 483 relevant posts remaining for analysis (Figure 2). Due to time constraints, there was no pre- and post-announcement analysis conducted for Announcement-2.

### 3.2. Online Activity

Post-Annoucement-1 results revealed a 1779% increase in online activity when compared to the pre-Annoucement-1 period, with posts/day average also increasing from eleven to 204. Announcement-2 showed an increase of 289%, with an average of 82 mentions/day when compared to the previous month. Announcement-1 Twitter activity returned to pre-announcement levels within four days, whilst Announcement-2 levels took longer than the weeklong analysis period to return to original activity levels. When comparing tweet-type breakdown between Annoucement-1 and -2, there was a noticeable difference. Whilst both tweet-type breakdowns show most of the Twitter activity being attributed to retweets, there was a greater proportion of original tweets within the Announcement-2 activity.

### 3.3. Author and Content Influence

The Meltwater analysis was supported by that from InfraNodus; both conveying the most prominent features of discussion, authors, and the prevailing themes within each announcement network (Table 3). The media activity that surrounded these three periods shows a distinct change from pre-Announcement-1, where anti-badger culling was the most prominent sentiment, with sporadic conversations regarding the commencing badger vaccination programs. Post-Annoucement-1 showed further support for the end of badger culling with an increased sense of urgency, and contained more posts commenting positively on badger vaccination rather than cattle vaccination. Announcement-2 showed the greatest support for cattle vaccination.

Meltwater analysis shows the change in the top key words, hashtags, and Twitter authors across Announcements- 1 and 2. InfraNodus was used to ascertain the change of the most influential elements within each category across the two announcements. Influential elements refer to the words that most commonly link conversations, topics, and authors within the digital and social media posts over the announcement timeframes. There was a considerable difference in top Twitter authors when comparing pre- and post-Announcement-1 periods, as individuals from the public with fewer followers were prominent initially (Table 3). However, in post-Announcement-1 the national news groups were the main disseminators and influencers of discussion—with the majority preferentially leading with posts signifying the end of the badger cull, rather than the potential for the cattle BCG vaccine. This content is especially important when observing the stark contrast in the number of Twitter followers and thus, social reach. For example, at the time of analysis, The Guardian had 9,000,640 Twitter followers, whilst Sky News had 5,534,464 and the CEO of the Badger Trust had just 23,368. By comparison, the digital and social media post content of Annoucement-2 focused heavily on the cattle BCG vaccine and introduction of a test to enable differentiating infected from vaccinated animals (DIVA) [21]. This again was supported by InfraNodus and the identification of the most influential elements to be ‘trial, bovine, cattle, vaccine’ (Table 3); however, it was not just the content that was notably different, but the authors as well.

### 3.4. Sentiment and Trend Analysis

**Figure 3:**
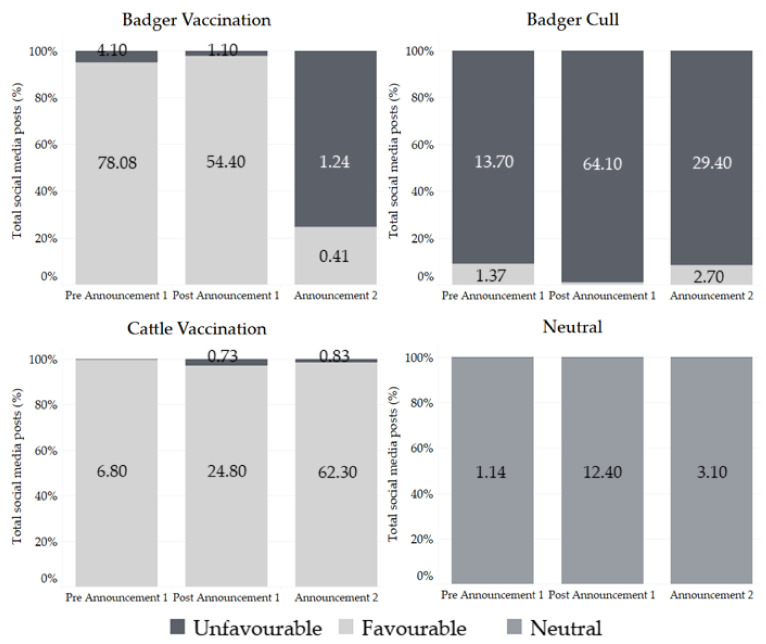
Bar graphs demonstrating the percentages of the total number of relevant digital and social media posts that were categorized as either unfavorable, favorable or neutral in reference to the topics of badger vaccination, badger culling, cattle vaccination, or neutral for the periods: pre-Announcement-1; post-Announcement-1; and Announcement-2.

Thematic analysis using Infranodus revealed changes between the categories that were represented as the ‘noisiest’ in each time-period. Figure 3 demonstrates the change in percentage of favorable and unfavorable digital and social media posts within the three categories: badger cull, badger vaccination, cattle vaccination, in comparison to the neutral category. Figure 3 shows that unfavorable posts towards the badger cull increased from 13.7% to 64.1% between pre- and post-Annoucement-1, whilst unfavorable badger cull posts represented 29.4% of Annoucement-2 posts. Badger vaccination lost support over each announcement period, falling from 78% to 54.4% to 1.2% respectively (Figure 3). Conversely, cattle vaccination conversations were mostly favorable and gained support, increasing from 6.8% to 24.8% after Announcement-1.

A notable data overlap revealed that 26% of digital and social media posts were found to discuss both badger culling in an unfavorable manner, as well as badger vaccination in a favorable manner within the post-Announcement-1 period. A 19% discussion overlap was also found, with digital and social media posts containing anti-badger cull sentiments as well as both badger and cattle vaccine favorable sentiments within post-Announcement-1. This increase in unfavorable sentiment towards the badger cull also incorporated more emotive language by several users; creating a greater sense of urgency within their hashtags e.g. #sensless, #inhumane and #stopthecullnow (Table 3) (Figure 3). Within Announcement-2, a substantial overlap of 47% was identified between posts that were favorable towards cattle vaccination and unfavorable towards badger culling. Interestingly, there is also a veterinary presence in Announcement-2 that was absent in Announcement-1, with the Vet Times UK featuring as a top Twitter author, and #cattlevets as a top hashtag (Table 3), which coincided with increased sentiments favoring the cattle vaccine.

**Figure 4:**
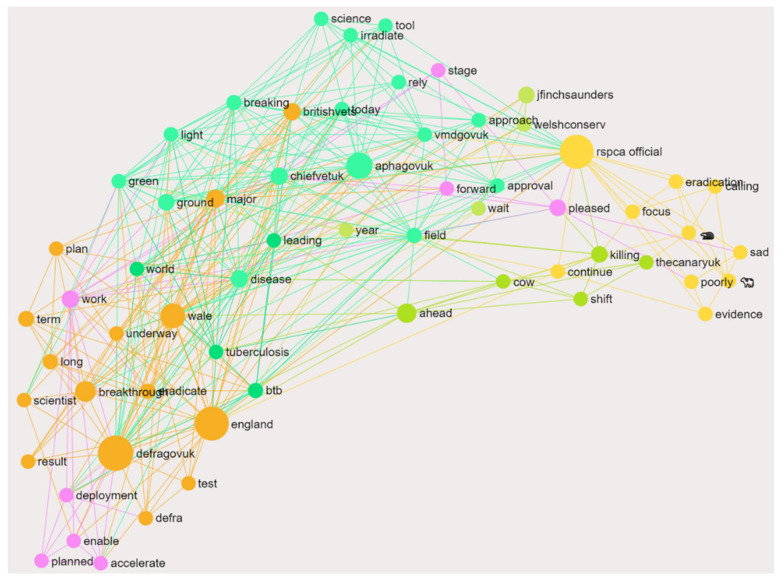
InfraNodus graphical visualization of the communication gap between the cattle vaccine favorable (CVF) for Announcement-2 and post-Announcement-1. Data present in Announcement-2 CVF, but absent in post-Announcement-1 CVF. Different colors represent different topical clusters that are connected, whilst lines show the connections between different words and clusters. Each word is represented by a node, with the node size changing depending on its level of influence on other words around it. For example, in this social network analysis, defragovuk was absent in Announcement-1, but present in Announcement-2 where it has a large orange node. This shows it is highly and diversely connected to other words and therefore has a greater overall influence on them and, upon detailed analysis, was found to influence the positive sentiment directed towards the bovine tuberculosis (bTB) cattle vaccine.

Conversation clusters supporting the cattle vaccine that were absent in post-Announcement-1 but present in Announcement-2 were identified via InfraNodus (Figure 4). There was a considerable difference between the two periods, with post-Announcement-1 missing 81% of the cattle vaccination favorable content present within Announcement-2. The main subjects of this absence in the initial announcement were shown to be influential authors; defragovuk (Department for Environment, Food and Rural Affairs), rspca_official (Royal Society for the Prevention of Cruelty to Animals) and aphagovuk (Animal and Plant Health Agency, APHA) (Table 3) (Figure 4). Twitter handles, including Britishvets, Chiefvetuk and jfinchsaunders, were noted to be influential and are important narrative shifting authors whose sentiment were also absent in Announcement-1 (Figure 4).

Using the InfraNodus comparison tool, the data absent in Announcement-1, but present in Announcement- 2 can be used to reveal latent topical brokers that had an unusually high rate of influence in comparison to their frequency; meaning that although these nodes do not appear as often as the most influential nodes, they are important narrative shifting points. The main examples of Announcment-2’s latent topical brokers include aphagovuk, defragovuk, chiefvetuk, rspca_official and jfinchsaunders; all of which were absent in Announcement-1 (Figure 4). These nodes also show an increased ability in bridging the structural gap in communication within Announcement-2 as there was increased positive sentiment directed towards the cattle vaccine, in comparison to Announcement-1. The structural gap in Figure 4 highlights important areas, which if linked by effective communication, have the potential for connecting and influencing both people and ideas within this social network. It is theorized that this improved connection will heighten overall public understanding of the government strategy.

## 4. Discussion

This study aimed to reveal the change in online response towards revisions to the government’s bTB eradication strategy for England by interpreting media ‘noise’ both before and after their announcement. We also aimed to identify any public misunderstandings resulting from communication gaps, as well as gain an understanding of the benefits and limitations of digital and social media monitoring platforms to gauge public perception. Our ‘online’ status as society means that social media use is ever-increasing, with an ever-broader audience. The importance of understanding public views of government announcements is vital to assess public confidence in policy strategy and may provide insight into gaining further support for both current and future government policies by bridging these communication and information gaps. To this end, Meltwater provides an automated media listening tool that can aggregate and analyze vast amounts of data in real-time, further commending its use for surveillance to understand how a government policy is being perceived and what conversational topics surround it. The ability to analyze social data provides a greater understanding of whether key messages within a government policy are resonating with the public or are being lost in the ‘noise’ of other conversations.

We detected an important surge in communication activity across each announcement period, with the controversial topic of the badger cull taking precedence over the cattle vaccine after Announcement-1 and vice versa after Announcement-2. Tweet-type breakdown showed an increased prevalence of original tweets within Announcement-2 communications. Original tweets are suggestive of a greater interaction with the topic as a larger number of Twitter users were adding their own views or questions to the original author’s dialogue, rather than merely retweeting the content alone. Retweets are indicative of a more rapid interaction with the information shared, and whilst this is an efficient way of sharing ideas and crediting authors, it could suggest less investigation into tweet content. Announcement-1’s retweet prevalence may be due to the dominating discussion of the controversial badger cull, which produced a polarized set of views that were either favorable or unfavorable, with very few digital and social media posts showing ambiguity. Whilst the ratios of tweet-type breakdown are limited due to differing periods of data collection for each announcement; Announcement-2, which showed increased support for the cattle vaccine, also demonstrated greater public interaction and clarification of tweet contents. This may be suggestive of a greater investment of understanding into the topic as a larger percentage of media users incorporated their cattle vaccine favorable views with their badger cull unfavorable views, inferencing the cattle vaccine as the definitive end to badger culling. Despite Announcement-2 not reaching the same noise levels as the previous announcement, this increased level of public interaction may explain why it took longer to return to original activity levels in comparison to post-Announcement-1.

Considering prior media attention mainly referencing the contentious subject of the badger cull rather than the cattle vaccine, it is unsurprising that this theme initially took precedence and represented most of the ‘noise’ created by digital and social media posts after Announcement-1. There are likely numerous reasons for this focus on ‘phasing out’ of badger culling rather than on the vaccination programs. First, badger culling has been a controversial topic since its introduction in 2013, resulting in widespread media coverage. We found animal welfare groups, such as the RSPCA, were strong influencers, with statements to save the ‘iconic’ British species gaining great traction among the British public. Second, due to the prominence of anti-culling discussion prior to this report, it is unsurprising that badger culling also took online precedence over vaccination. Third, the majority of news-related reports led with the announcement of an end to badger culling, followed by the progression to vaccinations, e.g., ‘Badger culls to be phased out in favor of vaccinations, Government announces’ [22]. News headline structure is created to optimize relevance and likely influences reader perception of the news [23]. As such, the primary statements leading with the announcement of an end to badger culling, which are followed by the mention of vaccinations will impact reader assessment of the government policy, with vaccinations taking more of a ‘back seat’ after Announcement-1. This reader perception was significantly altered upon the Guardian’s publication of the headline: ‘Bovine TB vaccine trials get go-ahead in England and Wales’ [24]. The greatest difference being the absence of the badger cull theme, directing reader focus solely to the bTB vaccine. Interestingly, the subheading did incorporate it [24]; ‘Scientific breakthrough could lead to phasing out of badger culling to tackle disease’, delivering overall support to the cattle vaccine trials, with the notion that it will bring an end to badger culling. A government announcement made after our analyses were completed that Natural England has licensed and authorized eleven new badger control areas to begin operations in 2020 and has authorized license holders to resume operations in 33 existing badger control areas in 2020 [25] has been viewed as reneging on the promise of phasing out intensive culling of badgers [26] and created further media noise beyond the scope of this study. These findings suggest that the news media may have an agenda setting influence in framing the public’s perceptions around badger culling and cattle vaccination. Further research should evaluate the extent of this influence by comparing digital and social media listening to surveys of public opinions and perceptions.

The influential capacity of online articles is dependent on the author’s social reach, which is dictated by their number of followers. Furthermore, the ‘noise’ and activity levels for each announcement corresponds to the social reach of the most prominent authors, with news platforms, such as Sky News and the Guardian, representing top Twitter authors for post-Announcement-1 and Announcement-2, respectively. The digitalization of these traditional media platforms may not only be influential due to their social reach, but also due to their perceived credibility by readers, and the view that online and printed newspapers contain the same content [27]. Announcement-2’s focus on the cattle vaccination field trials was clearly highlighted in digitalized news sources, but more notably, communications derived from direct government sources, including Defra and APHA. These sources of information were previously absent in Announcement-1 and appear to be contributing factors (along with the RSPCA) to the increased media attention on cattle vaccination. A distinction between the two announcements is the veterinary presence within Announcement-2, with the Vet Times UK featuring as a top Twitter author, and #cattlevets as a top hashtag (Table 3). A national survey of over 2000 people revealed that 94% of people trust veterinarians [28]. This level of public perception and confidence strengthens the idea that the public may be more inclined to endorse the use of cattle vaccines if they are recommended by veterinarians.

Qualitative analysis was performed on the content within the digital and social media posts collected to assess the key topics of discussion within each period. Unsurprisingly, the pre-Announcement-1 period was the quietest in terms of both online activity and variation of themes conversed, likely due to the lack of news publications. Emphasis was placed on the preparation for commencing badger vaccination programs. Support for the badger vaccine was present on the notion that its use was to ‘save badgers from being culled’. Some confusion was noted around the use of the badger vaccine and its efficacy to protect badgers from bTB, with some favoring the use of the cull to remove the ‘major reservoir’ for bTB as the vaccine was perceived as ‘ineffective’. However, the central theme was focused on the protection of badgers and wildlife from bTB and culling through use of vaccination (Table 3) (Figure 3).

A notable change in emphasis was detected in the post-Announcement-1 period, with the celebration of the end of badger culling being ‘in sight’ situated firmly at the forefront of discussion with support from wildlife trusts. Greater ambiguity was noted with the suggestion of a multi-faceted approach with ‘cull’, ‘controversial’ and ‘vaccines’ representing the top keywords. Surprisingly, the cattle vaccination topic generated the least noise, despite the increased support and positive sentiments. This is likely due to the contentious topic of badger culling dominating discussion, with most users focusing on the end of badger culling and not the reasoning behind it—a notion supported by the increased use of retweets for a more rapid response and offering of support without delving into the broader conversation. InfraNodus revealed a structural communication gap of ‘cattle, protect, phase and bovine, vaccine, cull’ between posts unfavorable to the badger cull and posts favorable to the cattle vaccine. This is suggestive that a communication bridge between these topics would improve public understanding of the links between these two categories and how they relate to one another. This communication gap was notably improved upon in Announcement-2, whereby public perception of the bTB vaccine was better integrated with the insight that its use would be imperative for end of badger culling.

Interestingly, the digital and social media posts that displayed the greatest ambiguity during Announcement-1 stemmed from farming communities; with the main apprehensions being centered around financial and time management concerns regarding badger vaccination, badger-responsibility for bTB spread, government policy mistrust, lack of evidence for badger vaccine efficacy and reliance on evidence demonstrating that ‘culling works’. This overlap changed significantly after Announcment-2, with badger vaccination becoming marginalized to the cattle vaccine favorable category, which contained a 47% overlap with the badger cull unfavorable category.

Announcement-2 was the noisiest time-period for the cattle vaccination category, with most posts being favorable towards the field trials (Figure 3). Positive themes discussed within the posts favorable to cattle vaccine, suggested using the bovine BCG vaccine as an end to badger culling. Support for the cattle vaccine was bolstered by the suggestions that the use of an effective bTB vaccine would reduce cattle slaughter number and the need for ‘such rigorous food safety checks’. Some misunderstanding was uncovered, with wildlife supporters inferring that if badgers have all been vaccinated, and cattle are still being diagnosed with bTB, then badgers were not the cause of infection.

A distinct confusion was noted regarding the detail that this was not the release of a novel cattle vaccine, but the development of the innovative DIVA test. This misunderstanding is important to remedy and signifies an area that requires further clarification in forthcoming announcements, as it may incite vaccine hesitant individuals to induce further controversy over a ‘novel vaccine’, an unnecessary misunderstanding that would be detrimental to the support of the government’s strategy. A quieter theme identified was a correlation between those who believe that whilst vaccination in cattle is positive progress, there is still significant need for badger culling to reduce the wildlife reservoir of the disease.

Negative themes from Announcement-2 included lack of support for the badger vaccine, with most suggesting it was ‘not effective’. Despite the overwhelmingly positive response for the cattle BCG vaccine there were several hesitancy-based discussions surrounding its use. The main concerns identified were largely comparable to post-Annoucement-1 and featured cattle vaccine efficacy, the period until it is available for use, financial implications, and the compatibility of vaccination with beef exports. BCG vaccine hesitancy regarding failure to induce protective immunity and prevent bacterial spread, and merely slowing pathogenic course was a prominent conversation.

Study limitations were mainly because of analysis timing, which was situated within the period of the coronavirus (COVID-19) pandemic. Naturally, COVID-19 received a considerable amount of digital and social media attention [29], with the mention of vaccines proving to be problematic within Annoucement-1 (Figure 1a). This was remedied by further tailoring of the Boolean search for Announcement-2 (Figure 1b). As only one researcher performed data categorization and analysis and no inter-rater reliability procedure was performed, this analysis was dependent on researcher subjectivity and interpretation of digital and social media content. Insights into the opinions within the digital and social media posts are based on reader observation and analysis of the social conversations, thus, to minimize bias, inclusion and exclusion criteria were used for categorization (Table 1). Digital and social media posts extracted from Meltwater are not representative of the UK population’s perceptions but focuses on media ‘noise’ that refers to online exposure and should be interpreted as such. Furthermore, content from private users is not accessible, consequently, information is revealed at the individual’s discretion. No quantitative analysis was performed due to the inability to assign both pre- and post-Announcement-1 and Announcement-2 participants, resulting in unpaired and independent categories.

## 5. Conclusions

The importance of understanding the role of both digital and social media and its influence on government policy has never been greater. Real-time digital and social media monitoring will be an important future tool to minimize the effects of misinformation and instead provide targeted communication, e.g., with identified vaccine-hesitant groups to gain public support for future government vaccine initiatives, including for COVID-19. To our knowledge, we are the first to apply this approach to the vaccination of animals against a zoonotic disease: bTB.

By using media monitoring we were able to identify and categorize public perception and matters of policy concern surrounding bTB that revealed the importance of exposure to concise and digestible information from reputable sources. This insight was further supported by the combined release of statements by both government, public health, and veterinary-backed platforms, which significantly improved public response towards a cattle vaccine for bTB. Digital and social media monitoring also revealed the importance of follow-up announcements in expeditiously addressing areas of public hesitancy to bolster policy support. Sentiment and trend analysis unveiled areas of government communications that required further clarification and/or greater media attention; a crucial action that may strengthen public and government relations in the future. In our case, this was exemplified in the finding that focused explanation that the bTB-free strategy does not involve the development of a new cattle vaccine, but rather validation of a DIVA test, should reduce policy misinformation and improve public clarity and support. Furthermore, additional explanation of policy implementation regarding timeframe, financial and trade implications may enhance public understanding of the bTB-free strategy, which in turn, would make policy acceptance more likely.

Our findings suggest that the news media may have an agenda setting influence in framing the public’s perceptions around badger culling and cattle vaccination. Further research should evaluate the extent of this influence by comparing digital and social media listening to surveys of public opinions and perceptions. More generally, future research of this nature would benefit from automated processes for the coding of posts. This would make the process significantly less time-consuming and allow for more real-time response by government. Such automated coding has been shown to be feasible in identifying stances towards vaccination during pregnancy by using transposer-based machine learning methods [30].

## Figures and Tables

**Table 1:** Digital and social media post categorization.

Category ^1^	Abbreviation	Definition
Neutral	NE	Post with content or sentiment towards vaccination or culling that is neither favorable nor unfavorable, expressing balanced, unbiased opinions or factual statements
Badger Cull Favorable	BCF	Posts displaying a positive sentiment or favorable views towards badger culling, for example encouraging the continued use of badger culling and reporting scientific support regarding the badger cull
Badger Cull Unfavorable	BCU	Posts displaying a negative sentiment or unfavorable views towards badger culling. Content could include requests to stop badger culling, support for the ‘Save Me’ movement to protect badgers from culling, positive sentiments towards badgers, discouraging badger culling (e.g., inhumane, lack of evidence of improving cattle TB rates, link to increased spread)
Badger Vaccine Favorable	BVF	Posts displaying a positive sentiment or favorable view towards badger vaccination, for example communicating the benefits of badger vaccination in optimistic or positive tones. Content could refute negative comments about the vaccine, encourage badger vaccination, or express positive views towards the current use of badger vaccines. Content could also include scientific reporting on the benefits of vaccination, including the humaneness of the approach
Badger Vaccine Unfavorable	BVU	Posts displaying a negative sentiment or unfavorable view towards badger vaccination, for example refuting positive comments about the vaccine, arguments against vaccination of badgers, concerns about the safety to badgers, cost of the vaccine or efficacy of vaccination
Cattle Vaccine Favorable	CVF	Posts displaying a positive sentiment or favorable view towards cattle vaccination, for example supporting the government announcement of vaccine trials, including its cost and benefits, ease of administration
Cattle Vaccine Unfavorable	CVU	Posts displaying a negative sentiment or unfavorable view towards cattle vaccination, for example concerns over cost, animal trade implications, length of time until vaccine is available, efficacy of cattle vaccine, or lack of scientific backing. It could also include concerns over interference with the Single Comparative Cervical Intradermal Test (SCCIT)
Ambiguous	AM	Posts containing both favorable and unfavorable sentiments towards badger culling/vaccination and/or cattle vaccination. Unable to decipher true intent of the individual
Unknown Relevance	UR	Posts that were inaccessible for evaluation or were in a foreign language
Irrelevant	IR	Posts that were not relevant to the topic. For example, Coronavirus-related

^1^ Category definition used for the classification of individual digital and social media posts on the Meltwater media monitoring platform.

**Table 2:** Numerical categorization of digital and social media posts.

Category	Pre-Announcement-1	Post-Announcement-1	Announcement-2
Badger Cull:			
Favorable	1	20	13
Unfavorable	10	1746	142
Total	11	1766	155
Badger Vaccine:			
Favorable	57	1,482	2
Unfavorable	3	30	6
Total	60	1512	
Cattle Vaccine:			
Favorable	5	674	300
Unfavorable	0	20	4
Total	5	694	304
Neutral:	1	337	15

**Table 3:** Prominent features of discussion, authors and the prevailing themes.

Category	Pre-Announcement-1	Post-Announcement-1	Announcement-2
**Meltwater Analysis ^1^**			
Top Key Words	bovine tb, training, summer	bovine tb, cull, vaccines, decision, disease, controversial	disease, bovine tb, tb vaccine trails, cattle vaccine, diva test
Top Hashtags	#badgermonday #wildlife #stopthecull	#badgercull #wildlife #stopthecullnow #sensless	#badgercull #science #btb #stopthecull #cattlevets
Top Twitter Authors	Individuals from the public e.g. CEO of The Badger Trust	Sky News (Sky UK Ltd., Isleworth, Middlesex, UK), The Daily Mail UK (Daily Mail and General Trust, London, UK), RSPCA official (Royal Society for the Prevention of Cruelty to Animals, Southwater, West Sussex, UK), BBC Bristol, Gloucester, Midlands, Cumbria, and BBC Country File (British Broadcasting Corporation, London, UK), Farmers Weekly (Farmer Weekly, Sutton, Surrey, UK), Farmers Guardian (Farmers Guardian Ltd., Preston, Lancashire, UK)	The Guardian (Guardian Media Group, London, UK), RSPCA (Royal Society for the Prevention of Cruelty to Animals, Southwater, West Sussex, UK), Defra (Department for Environment, Food and Rural Affairs, London, UK), ITV West Country (ITV plc., London, UK), Farmers Guardian (Farmers Guardian Ltd., Preston, Lancashire, UK), Vet Times UK (Veterinary Business Development Ltd, Peterborough, UK), APHA (Animal and Plant Health Agency, Addlestone, Surrey, UK)
**InfraNodus Analysis ^2^**			
Most Influential Elements	badger, @wildlifetrusts (The Wildlife Trusts, Newark-on-Trent, Nottinghamshire, UK), wildlife	badger, cull, vaccine, @wildlifetrusts, cattle	trial, bovine, cattle, vaccine, @defragovuk ((Department for Environment, Food and Rural Affairs, London, UK), @rspca_official (Royal Society for the Prevention of Cruelty to Animals, Southwater, West Sussex, UK), @aphagovuk (Animal and Plant Health Agency, Addlestone, Surrey, UK)

^1^ Meltwater analysis shows the change in the top key words, hashtags, and Twitter authors across Announcements-1 and 2. ^2^ InfraNodus was used to ascertain the change of the most influential elements within each category across the two announcements. Most influential elements refers to the words that most commonly link conversations, topics, and authors within the digital and social media posts over the announcement timeframes.

## Data Availability

No new data were created or analyzed in this study.
